# Religiosity and Spirituality and the Intake of Fruit, Vegetable, and Fat: A Systematic Review

**DOI:** 10.1155/2013/146214

**Published:** 2013-11-10

**Authors:** Min-Min Tan, Carina K. Y. Chan, Daniel D. Reidpath

**Affiliations:** Jeffrey Cheah School of Medicine and Health Sciences, Monash University, Sunway Campus, Jalan Lagoon Selatan, 46150 Bandar Sunway Selangor Darul Ehsan, Malaysia

## Abstract

*Objectives*. To systematically review articles investigating the relationship between religion and spirituality (R/S) and fruit, vegetable, and fat intake. *Methods*. PubMed, CINAHL, and PsycInfo were searched for studies published in English prior to March 2013. The studies were divided into two categories: denominational studies and degree of R/S studies. The degree of R/S studies was further analyzed to (1) determine the categories of R/S measures and their relationship with fruit, vegetable, and fat intake, (2) evaluate the quality of the R/S measures and the research design, and (3) determine the categories of reported relationship. *Results*. Thirty-nine studies were identified. There were 14 denominational studies and 21 degree of R/S studies, and 4 studies were a combination of both. Only 20% of the studies reported validity and 52% reported reliability of the R/S measures used. All studies were cross-sectional, and only one attempted mediation analysis. Most studies showed a positive association with fruit and vegetable intake and a mixed association with fat intake. *Conclusion*. The positive association between R/S and fruit and vegetable intake may be one possible link between R/S and positive health outcome. However, the association with fat intake was mixed, and recommendations for future research are made.

## 1. Introduction

Unhealthy diet is a major risk factor in the development of noncommunicable diseases (NCD), which are responsible for about 63% of deaths globally [1]. One of the main characteristics of a healthy diet is the regular consumption of a variety of fruit and vegetable, which is associated with a lower risk of some cancers, coronary heart disease, hypertension, and stroke [2, 3]. About 1.7 million deaths worldwide are attributed to a low fruit and vegetable intake [4]. In addition, about 14% of gastrointestinal cancers, 11% of ischemic heart disease, and 9% of stroke worldwide are also attributable to low fruit and vegetable intake [5]. Another important dietary factor related to health is fat intake. A high fat intake is associated with higher risk of coronary heart disease, diabetes, and cancers, the common NCDs [6–8]. 

The World Health Organization recommends a daily intake of at least 400 g (5 servings) of fruit and nonstarchy vegetable, and fat that is less than 30% of total dietary energy, of which less than 10% is from saturated fat and less than 1% from transfat [9]. However, due to urbanization and westernization, many countries that traditionally enjoyed high fruit and vegetable and low dietary fat consumption are moving towards a higher fat, lower fiber diet [10]. The global burden of NCD is predicted to increase further because of this global transition in lifestyles [10].

Research has shown that religion and spirituality (R/S) has a positive association with health [11]. About 80% of the studies looking at the relationship between R/S and health examined mental health [12], showing a positive association with wellbeing, self-esteem, and optimism [13–16]; lower scores of depressive and suicidal symptoms [17, 18]; and lower stress level [19–21]. Numerous studies have also reported a positive association between R/S and physical health, including an association with lower all-cause mortality and lower rates of diet-related diseases such as hypertension, cardiovascular diseases, and cancer [22–26]. 

The study of the relationship between R/S and health remains relatively novel. Initially it was not well accepted because it was thought that it is impossible (within a positivist framework) to study R/S scientifically. However, starting in the early 1990s, with the improvement of methodology, the study of religion and health has been increasingly recognized as a legitimate domain of scientific inquiry and is becoming more established [27]. Between 2000 and 2010, at least 21,000 quantitative studies examining the relationship between R/S and health have been published [28], covering a wide range of health outcomes and behaviors. 

In the research of R/S and health, one of the major issues is defining religion and spirituality in a way that supports their measurement. Historically the notions of religion and spirituality have often been used interchangeably; however, recently there has been a trend towards distinguishing the two concepts [29]. Broadly, religion includes “beliefs, practices, and rituals related to the Transcendent or the Divine” [30], while spirituality is concerned with the “connection to that which is sacred, the transcendent” and also “a search for the transcendent and the discovery of the transcendent” [28]. Religion tends to convey a negative impression that it is related to organized religion and theological rigidness, while spirituality is viewed more positively and is associated with personal experience of the transcendent. However, Koenig recommends the use of spirituality in the context of religion, that is, those who are spiritual are “deeply religious” [30]. 

The most commonly used R/S measure has been the single-item measuring religious attendance because of its “ease of use” [31]. In most of the studies that have used a religious attendance measure, most have also found that it is positively associated with better health outcomes [32]; for example, it has been associated with lower mortality rates [33, 34], better adoption of health behaviors [35, 36], more life's satisfaction [37], and a lower prevalence of hypertension [24]. However, in many studies, R/S data were collected as part of a larger study, and this can be a drawback [38, 39]. It is generally accepted that R/S is a multidimensional construct [40], which means that a single-item measure such as religious attendance will be insufficient to capture all dimensions except possibly in the most general sense. This also limits insights that can be gained about the relationship between R/S and health. Recently, more specific scales have been developed to measure different dimensions of R/S [40]. 

Religion is considered important to many people around the world. One recent survey estimated that 51% of the population in the world believe in god(s) [41]. Another survey conducted in 143 countries found that the majority of people, especially those from developing countries, reported that religion was an important part of their lives [14]. 

One of the proposed mechanisms by which R/S benefits health is through the adoption of religious practices that are also health-promoting [42]. Many religions view the human body as sacred and include specific prohibitions against unhealthy behaviors, which are considered irreverent and not only harmful physically but also spiritually. This view, however, needs to be tempered by the fact that some religious adherence *may* also result in poorer health outcomes, such as extreme asceticism. Notwithstanding that, numerous studies have shown that R/S is negatively associated with many harmful behaviors such as smoking [43, 44], alcohol drinking [45, 46], substance abuse [47, 48], and risky sexual activities [49, 50] and positively associated with good health behaviors such as the use of preventive health care services [51, 52], physical activity [36], and seat belt use [53]. 

Certain health practices are endorsed and encouraged by most religions, such as healthy eating. In fact, most religions have specific dietary guidelines regarding what food to eat or avoid. These guidelines fall into two categories. The first category involves “a temporal abstinence from all or certain foods (fasting)”—the majority of religions have fasting guidelines, for example, Muslims fasting during Ramadan and oriental orthodox Christians fasting before Holy Communion. The second category relates to “stable and distinctive dietary habits that differ from the general population”; for example, Muslims consume halal meat and Jews consume kosher meat [54]. The main purposes of these dietary guidelines are for spiritual advancement. 

R/S might encourage the consumption of fruit and vegetable and discourage fat intake (especially animal fat) because of specific doctrines of a particular religion. For example, the teaching of Ahimsa (do no harm) in Mahayana Buddhism and Hinduism encourages their adherents to be vegetarians in order to cultivate compassion, since eating animals requires slaughtering. Nonvegetarian food is considered impure and could hinder one's spiritual development [55]. The Seventh-day Adventists are encouraged to be vegetarians, as part of a religious duty to maintain a healthy body [56]. Even in religions that do not have specific dietary guidelines or restrictions, the teaching that the body is sacred might encourage the adoption of healthier behaviors, including a healthier diet.

The long term dietary practices required by certain religions could be a protective factor in preventing diet-related diseases. For example, the Seventh-day Adventist Church and the Church of Jesus Christ of the Latter-day Saints both encourage their believers to consume more fruit and vegetable and less fat. There is evidence from observational studies that Adventists, Mormons, and adherents of religions with strict dietary guidelines have healthier diets, better physical health, and longer lifespans than the general population [57, 58]. 

Notwithstanding the importance of food in many religions, there is a surprising scarcity of research on the relationship between R/S and diet. According to the first edition of the Handbook of Religion and Health [59], the most comprehensive review about R/S and health to date, there were only seven studies on R/S and diet before 1990. The second edition of this Handbook (2012) reviewed 21 studies about R/S and diet between 2000 and 2012. Sixty-two percent showed a positive association; that is, a higher measured R/S is associated with a healthier diet. 

A few other reviews also identified generally positive associations between R/S and a healthier diet. Groen and van der Heide [60], for instance, reviewed the role of dietary cholesterol in the development of atherosclerosis and coronary thrombosis among adherents of different religions. They found that Jews and vegetarian Trappist monks have a lower cholesterol level than the comparable groups. Shatenstein and Ghadirian [61] reviewed the differences in health behaviors, including dietary practices, among different religious groups. In another review, Sarri et al. [62] examined religious dietary practices and physical health among Muslims, Seventh-day Adventists, orthodox Christians, Jews, Buddhists, and a few other religions. There was an inconsistent finding about the influence of Ramadan fasting on physical health among Muslims and an overall positive relationship between religious dietary practices and health in other religions.

However, to date, there has been no review that examined the relationship between R/S and specific dietary intake. The past reviews have only examined R/S and diet in general. Thus, the purpose our review was to address this gap and systematically review the relationship between R/S and fruit, vegetable and fat intake. We hypothesized that R/S was positively associated with fruit and vegetable intake, and negatively associated with fat intake. 

## 2. Methods

### 2.1. Search Strategy

PubMed, CINAHL, and PsycInfo were searched by using two categories of key terms: religious key terms (religion, religiosity, religiousness, and spirituality) and dietary key terms (diet, food, food habits, health behavior, food preferences, eating, nutritional status, fruit, vegetable, fibers, and fats). The Boolean operator “OR” was used to combine key terms within each category, and “AND” to combine both categories. In PubMed database, the “NOT” operator was also used to eliminate studies related to clinical trials, fasting, reviews, systematic reviews, case reports, editorial, and comment. The full search strategy can be obtained from the authors. 

### 2.2. Inclusion Criteria

To be eligible for inclusion, a paper had to fulfill the following criteria.

1. The research analyzed the direct association between at least one quantified R/S measure and at least one quantified measure of fruit and vegetable or fat intake. Thus, qualitative studies and case studies were excluded.
2. The paper was published in English and in a peer-reviewed journal before 1 March 2013.

### 2.3. Exclusion Criteria

A paper was excluded if

1. R/S and fruit, vegetable, and/or fat intake were included but their relationship was not examined directly (e.g., parents' R/S and children's intake)
2. only overall health/dietary behavior was assessed but not fruit, vegetable, and/or fat intake specifically
3. R/S was included as part of the measure of another variable (e.g., social support) but the direct relationship between R/S and fruit, vegetable, and/or fat intake was not assessed;
4. the focus of the paper was fasting and/or eating disorders;
5. the paper examined *only* serum levels of nutrients and not direct intake. Serum level or biomarkers of nutrients might not be an accurate indicator of fruit, vegetable, and fat intake since the nutrients could be obtained from supplements;
6. the paper examined fiber intake but *did not* specify its dietary source as fruit and vegetable. Fiber could be obtained from supplements and nonfruit or vegetable food source such as grains.

The references and bibliographies of the papers were also examined to identify other relevant articles. Previous reviews of the relationship between diet (generally) and R/S were also examined [28, 59, 61, 62], and in one case the review author was contacted for his list of papers, which were not detailed in the review itself.

## 3. Analysis

The frequency and types of fruit, vegetable, and fat intake measures used were first examined. The measures were categorized into dietary records, 24-hour dietary recall, food frequency, brief dietary assessment methods, and dietary history. A dietary record is a detailed record of all food and drinks consumed over a period of time by a respondent; in 24-hour dietary recall, a respondent is asked about the food and drinks he/she consumed during the past 24 hours; a food frequency questionnaire is a list of commonly consumed food that could be selected by respondents; brief dietary assessments are used to estimate the intake of a nutrient or a type of food but do not assess overall diet; dietary history assesses dietary patterns over time [63]. 

The studies were divided into two categories: (1) denominational studies that compared fruit, vegetable, and/or fat intake between members of different religions, or denominations within the same religion, or between a religion with the general population and (2) degree of R/S studies that examined the degree of R/S and its association with fruit and vegetable and/or fat intake. The two categories were analyzed separately. 

The analyses of degree of R/S studies were guided by Rew and Wong [64] and Wong et al. [65]. First the categories of R/S measures were analyzed. The classification scheme was based on Wong et al. [65], which is a modification of Hackney and Sanders [66]. There are six categories: institutional (social and behavior aspects of R/S, e.g., attendance and social support), ideological (R/S beliefs, e.g., importance of religion), personal devotion (personal and internalized devotion, e.g., private prayer), existential (measures that are spiritual but not religious, e.g., spiritual wellbeing), multidimensional (examined more than one category of R/S), and generic (e.g., one-item measure that asks about how religious are the respondents) [65]. The relationships (positive (+), negative (−), mixed, or none) between R/S measures and fruit, vegetable, and fat intake were identified. 

The quality of R/S measures was assessed by examining whether their validity and reliability were reported. The number of studies that used single-item measure was also examined. Many R/S and health studies relied solely on the use of single-item measure of religious attendance, which has its limitations in health research [38]. The quality of research design (control for covariates, utilization of longitudinal data, and investigation of mediators) was assessed [64]. The studies were also categorized based on their reported relationship between R/S and fruit, vegetable, and fat intake. 

## 4. Results

**Figure 1:**
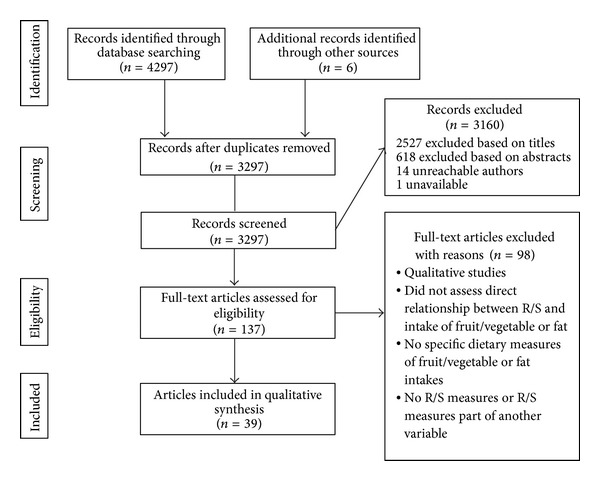
Articles selection process.

Out of the 3298 potentially relevant papers identified by the database search strategy, 32 papers fulfilled the inclusion criteria. An additional seven papers were obtained through cross reference of included papers and previous reviews.  Figure 1 shows the flowchart of article selection process. All of the 39 studies were cross-sectional. There are 14 denominational studies that do not contain other R/S measures, 21 studies that examined only degree of R/S, and four that included both denominational differences and degree of R/S. See Table 1 for the table of characteristics of the 39 studies.

The majority (77%) of the studies were conducted in the United States. Five were conducted in other Western countries (two in Australia (5.1%), and one each (2.6%) in Scotland, Slovakia, and Canada), two in Israel (5.1%) one in Japan (2.6%), and one in South Korea (2.6%). Four studies included only female samples. Eleven studies were race-specific; seven examined African Americans, two examined non-Hispanic Whites, one examined Koreans, and one examined Japanese. Thirty-two of the studies (79.4%) included samples that were predominantly Christians. Four studies examined Jews and three examined Buddhists.

### 4.1. Assessment of Dietary Intake

Out of the 39 studies, 12 examined fruit, vegetable, and fat intake, 14 examined only fruit and vegetable intake, and the other 13 only examined fat intake. 

Table 3 shows the categories of dietary assessments of fruit, vegetable, and fat intake. Among the five categories of dietary assessment methods, brief dietary assessments were the most used, followed by food frequency. The most used brief dietary assessment was the Fat- and Fiber-Related Behavior Questionnaire [67], which was included in four studies, followed by the National Institute's 5-A-Day Survey [68], which was included in three studies. Three studies used more than one dietary assessment method, and two used more than one brief dietary assessment. 

### 4.2. Denominational Studies

A total of 18 studies were analyzed. Eight (44%) of them compared Seventh-Day Adventists with the other denominations (Catholics, Methodists, and Mormons) or non-Adventists, three compared fruit, vegetable, and fat intake, three compared only fruit and vegetable intake, and two compared only fat intake. Among the six studies that compared fruit and vegetable intake, three (50%) showed that Adventists consumed significantly more fruit and vegetable than members of other denominations and non-Adventists, one (16.7%) had positive but nonsignificant association, one was nonsignificant in vegetable intake but significant in higher fruit intake, and one showed that Adventists consumed less deep fried vegetable.

Five studies compared total fat intake between Adventists and non-Adventists. Two (40%) showed that Adventists consumed less fat. One showed that Adventists consumed less fat when comparing total fat in grams, but similar amount of fat as non-Adventists when comparing the percentage of energy from fat. Another study showed that Adventist females consumed more fat. One showed no significant relationship. These five studies also examined the intake of saturated fat, and all found that Adventists consumed less saturated fat, even though their total fat intake was similar to those of non-Adventists. Three of the studies also compared the intake of unsaturated fat. Two found no significant relationship, and one found that among Adventists, the vegetarians consumed more unsaturated fat than Mormons, but Adventist omnivores consumed less saturated fat than the Mormons. Among the studies that compared unsaturated fat intake, two also looked at the polyunsaturated fat and saturated fat (P : S) ratio and found that Adventists had a higher P : S ratio.

Two of the denominational studies examined Jews. One compared the Jews with the general population in Italy and found that there was no difference in fat intake between the two groups. However, Jews consumed more animal fat than the general population. Another study compared two Jewish Hassidic sects and found that Lubavitcher Hassidim consumed more cooked fruit. 

There are two denominational studies that examined fat intake in Buddhists. The study that compared Japanese Zen Buddhist monks with the general population in Japan found that Zen Monks consumed less total fat and saturated fat, more unsaturated fat, and had a higher P : S ratio. However, another study that compared Buddhist nuns with Catholic nuns found no significant difference in total fat and unsaturated fat intake, but Catholic nuns consumed more animal fat.

One study compared Mennonites with the general US population and found that the Mennonites have higher total fat, saturated fat, and unsaturated fat intake. Another one study compared Catholics with non-Catholics in Scotland and found that Catholics consumed less pure fruit juice and Catholic males consumed less fruit and vegetable.

Four studies were multidenominational. One examined fruit, vegetable, and fat intake and found no significant relationship. Three studies examined fat intake only. One found no significant association. One study found that religious denomination mediates fat intake. One showed that among females, conservative protestant and those who have no religious preferences consumed more fat than Catholics.

### 4.3. Degree of Religiosity Studies

A total of 25 studies were analyzed. The R/S measures were categorized into the six categories described in the previous section. 

Table 4 shows the categories of R/S measures and their association with fruit and vegetable intake. The most commonly used R/S measures are multidimensional (36.8%). Among the 19 R/S measures, eight (42.1%) showed a positive association with fruit and vegetable intake and another seven (36.8%) showed no significant relationship. Among the seven measures that showed no significant relationship, four of them showed evidence of a positive trend in the relationship between fruit and/or vegetable intake. 

Table 5 shows the categories of R/S measures and their association with total fat intake. The most commonly used R/S measures are institutional (40.7%). Among the 27 R/S measures, 15 (55.6%) showed no significant relationship between R/S and total fat intake, while seven (25.9%) showed a negative relationship. Among studies that show no significant relationship, five showed evidence of a positive trend, and seven showed evidence of a negative trend. 

In addition to total fat intake, three studies also examined saturated fat intake. Two studies showed no significant association. One examined the degree of orthodoxy among Jews and found that more Orthodox Jews consumed less total fat and saturated fat, more unsaturated fat, and have a higher P : S ratio. 

Only 20% (5 out of 25) of the papers reported the validity of R/S measures, and 52% (13 out of 25) reported reliability of at least one of the R/S measures. Of the 12 studies that do not report reliability, three of them utilized a single-item measure of attendance. The majority of the studies (88%) controlled for covariates such as age, gender and years of education. All the studies were cross-sectional. Only one study investigated the mediator between R/S measures and healthy behaviors. However, since the mediator (self-assurance) was not associated with the intake of fruit and vegetable in the study, no further mediation test was carried out.

Table 6 shows the number of studies categorized based on their reported relationship. Of the 17 studies that examined the degree of R/S and fruit and vegetable intake, R/S reported positive association with fruit and vegetable intake in about half (52.9%) of the studies and no association in 35.3% of the studies. As for fat intake, almost half (46.7%) of the studies reported no association, and an equal number (20%) reported positive and negative findings. 

## 5. Discussion

About half of the denominational studies compared Adventists and non-Adventists. Healthy eating is one of the major teachings in the Adventist Church; other Christian denominations (except the Church of Jesus Christ of the Latter-day Saints) do not emphasize healthy eating as much as the Adventist Church. Thus, it is not surprising to find that Adventists generally consumed more fruit and vegetable and less total fat and saturated fat than non-Adventists. Similarly in studies that compared Buddhist monks and nuns with non-Buddhists, because of the teaching of Ahimsa (do no harm), Buddhists monks and nuns are vegans, and again it is not surprising to find that they consumed less saturated fat or animal fat. A weakness of denominational studies is the assumption of homogeneity of dietary practices among the members within a denomination. Denominational studies only compared denominational differences as a whole and omitted the individual variation of R/S of members within a denomination. It is unknown whether this variation is associated with dietary intake. In addition, denominational studies are “likely to be confounded with region and the effects of socioeconomic status” [69], and almost all of the denominational studies in this review did not control for covariates. 

Four of the denominational studies included samples from various Christian denominations. Three of the studies found no significant relationship between religious denomination and dietary intake. The nonsignificant findings were probably due to the fact that respondents from various denominations interpreted questionnaire items related to R/S differently. 

Although a meta-analysis was not conducted due to heterogeneity of R/S measures and dietary measures, the present review on the relationship between degree of R/S and fruit and vegetable intake points towards a positive association; that is, a higher score of R/S is associated with higher fruit and vegetable intake. About half of the Christian studies showed a significant positive relationship with fruit and vegetable. This is consistent with the previous review that R/S is associated with a better diet [28]. The results of the present review also suggest that the regular consumption of fruit and vegetable may be one of the possible links between R/S and positive health outcomes. Other possible links include adoption of other health behaviors such as the no smoking and drinking; better social integration and social support from religious communities; higher self-esteem and personal efficacy among the more religious; better coping resources and behaviors; positive emotions from religious practice; and healthy beliefs [42]. 

Six of the 17 studies reported no association between degree of R/S and fruit and vegetable intake. All three studies that included only Jewish samples showed no association. This may arise because the dietary restrictions of Judaism only revolve around meat and animal products and not on fruit and vegetable. The consumption of fruit and vegetable is neither restricted nor encouraged. 

The findings for fat intake contradicted the previous review. Almost half of the studies reported no association, and an equal number reported positive and negative findings. The contradiction might be due to the fact that the previous review examined diet as a whole and not particularly fat intake. There are other studies which showed that R/S was positively associated with greater body weight [70] and obesity [71], both of which might be related to high fat intake. The proposed explanation of higher prevalence of obesity among religious people could be that religious community is more accepting towards obese people, rather than R/S itself being the cause of obesity [71]. Kim et al. [70] found that the positive relationship between R/S and greater body weight disappeared after controlling for health behaviors, particularly smoking. None of the degree of R/S studies in this review controlled for health behaviors and it is unknown whether similar attenuation effect was also found between R/S and fat intake. 

Most of the studies in this systematic review included samples that were from the USA and Western countries where Christianity is the predominant religion; only two studies were conducted in Asia and one in Africa, even though religion is considered important by most people on these two continents [14]. None of the studies examined Hinduism and Islam, the two major religions in the world besides Christianity. Only three studies examined Buddhists; however, they were Buddhists in the USA rather than in Asia, even though there is a higher percentage of Buddhists in Asia. 

The most frequently used dietary assessment methods were brief dietary assessments. However, they are crude estimates of dietary intake. For example, the Fat- and Fiber-Related Behavior Questionnaire does not report dietary intake per se but only an overall score of fruit and vegetable and fat intake. Dietary records are considered the “gold standard” of dietary assessment methods [68]. However, only two studies in this review utilized dietary records. No studies used dietary history, probably because they are cross-sectional and assessing dietary history is time-consuming. 

The present review also showed a diversity of R/S measures used. Even within a category (see Table 2), there was variation. For example, the R/S measures coded as “institutional,” defined as “measures that focused on the social and behavioral aspects of R/S” [65], included attendance and religious social support, which are two different concepts that warrant further categorization.

Because of the diversity of R/S measures and that different R/S measures show different effects in different populations, it was proposed that R/S should be treated as a multidimensional construct [69]. However, less than a third of the R/S measures included in the present review are multidimensional. 

Very little information was provided with regard to the psychometric properties of the R/S measures. In this review, only 20% of the papers reported validity and 52% reported reliability of at least one R/S measure. However, only three studies (out of 25) used single-item measures of religious attendance. The overall quality of the degree of R/S studies was mixed, most of the studies control for covariates, but none of them used longitudinal data and only one attempted mediation analysis. All the studies were cross-sectional; thus the inference of causal relationship between R/S and fruit, vegetable, and fat intake could not be established. In R/S and health research, there are very few experimental studies, and the wide use of cross-sectional data is another major drawback, in addition to lack of clear definition of R/S [39]. 

There are several limitations in this review. Only peer-reviewed studies that are published in English were included. This could be the reason why most studies in this review were from Western countries and included mostly Christian samples. Second, unpublished studies were excluded and this might lead to publication bias, since studies with significant results are more likely to be published. Nonetheless, the present review was the first that examined the relationship of R/S with specific dietary intake. 

The contradictory findings among the studies of degree of R/S point to the need for more studies that control for health behaviors, for example, smoking, and use more rigorous dietary assessment method. In addition, more studies are needed that include participants of other religions, especially those of Eastern traditions and from non-Western countries. There is also a need to use more rigorous R/S measures that are validated, reliable, and multidimensional. 

## 6. Conclusion

Overall, the denominational studies showed that religious denomination is significantly related to fruit, vegetable, and fat intake. Specifically, the Adventists consumed more fruit and vegetable and less fat than non-Adventists. However, the relationship between the degree of R/S and dietary intake is mixed. The results of this review suggest that future research on R/S and diet may help explain the possible mechanism between religion and health. Methodology more sophisticated than observational studies is required. Longitudinal study methodologies (while still often observational) may enhance our understanding of underlying mechanisms. As religion is important for many people and affects their diet, improved methodological quality of R/S and diet research will surely shed more light on this area.

## Figures and Tables

**Table:** (a) Denominational studies (Adventists versus others)

No.	First author (publication year)	Location	*N*	Population	Sampling method	Denominations	Dietary measures		Dietary assessment methods	Finding	Control variables
							F/V	Fat			
1	Alexander (1999) [72]	Denver, USA	94	Adults	Convenience	Adventists Catholics	*√*	*√*	FFQ	Adventists (i) more fruit/vegetable (ii) less fat (iii) less saturated fat (iv) less % energy from saturated fat Nonsignificant: (i) polyunsaturated fat (ii) % energy from polyunsaturated fat (iii) % energy from fat	Gender

2	Hunt (1988) [73]	LA, California, USA	290	Postmenopausal women	Convenience	Adventists Methodists	*√*		24 hr dietary recall	Adventists nonsignificantly more fruit and vegetable (*P* = 0.08)	None

3	Kent (2009) [74]	Melbourne, Australia	1054	Adults	Random	Adventists Non-Adventists	*√*		FFQ	Adventists more fruit and vegetable	Age

4	Kuczmarski (1994) [75]	North Carolina, USA	227	Adolescents	Convenience	Adventists Non-Adventists	*√*	*√*	FFQ	Adventists (i) males & females: more fruit (ii) females: more fats	None

5	Rouse (1983) [76]	Perth, Australia	293	Adults, 22–44 yrs	Convenience	Adventist (vegetarians & omnivores) Mormons (omnivores)		*√*	24 hr dietary recall	Mormon males more total fat & saturated fat intake than male Adventist vegetarians Polyunsaturated fat—Adventist vegetarians > Mormons > Adventist omnivores P : S ratio—low in SDA omnivores and Mormons	None

6	Sabaté (1990) [77]	USA	1765	School children, 1st–10th grades	Random	Denominational school—SDA or public	*√*		FFQ	Adventist school children more fruit/vegetable	No

7	Shultz (1983) [78]	Oregon, USA	23	Adults	Random	Adventists (vegetarians) Non-Adventists (nonvegetarians)		*√*	3-day dietary record FFQ	3-day dietary record: nonsignificant, though non-Adventists 18% more total fat FFQ (i) Non-Adventists more deep fried vegetable (ii) Non-Adventists more animal fat and more saturated fat	None

8	Fraser (1987) [79]	LA & Orange Counties, USA	320	Non-Hispanic Whites, 35–55 yrs	Random (Adventists) & convenience (non-Adventist neighbors)	Adventists Non-Adventists		*√*	FFQ	Adventists—less fat, less saturated fat, higher P : S ratio; linoleic acid none	None

**Table:** (b) Denominational studies (other religions/denominations)

No.	First author (publication year)	Location	*N*	Population	Sampling method	Denominations	Dietary measures		Dietary assessment methods	Finding	Control variables
							F/V	Fat			
9	Epstein (1956) [80]	New York, USA	415	Garment workers, >40 yrs	Random	Italian Jewish Others		*√*	24-hr dietary recall	Italians and Jews—no difference in fat intake Jews more animal fats	None

10	Glick (1998) [81]	Yates Country, NY, USA	149	Old order Mennonites	Unclear	Mennonite USA population		*√*	FFQ	Mennonites males & females more fat & oleic acid than USA sample Mennonite males—similar % calories from fat with USA sample	None

11	Kita (1988) [82]	Kyoto, Japan	36	Adults, 24–35 yrs	Convenience	Zen monks University students		*√*	24-hr dietary recall	Zen monks less fat intake & higher P/S ratio	None

12	Lee (2009) [83]	Gyeongbuk, Republic of Korea	85	Females—Buddhist nuns & Catholic nuns	Convenience	Buddhists Catholics		*√*	Fat in % kcal Total fat Plant fat Animal fat	No differences in fat in % kcal, total fat, plant fat Catholics more animal fat	None

13	Mullen (2000) [84]	West of Scotland, UK	985	Adults, >35 yrs	Stratified random	Catholics Non-Catholics	*√*		FFQ	Catholics—pure fruit juice Catholic males—less fruit and vegetable	Sex, occupational class

14	Shatenstein (1993) [85]	Greater Montreal, Canada	250	Hassidic families	Random	Hassidic sects—Outremont Hassidim & Lubavitcher Hassidim	*√*		Food frequency & food habits list 2-day food record	Lubavitcher Hassidim more cooked fruit	None

**Table:** (c) Denominational + degree of R/S studies

No.	First author (publication year)	Location	*N*	Population	Sampling method	R/S measures	Dietary measures		Dietary assessment methods	Finding	Control variables
							F/V	Fat			
15	Kim (2004) [86]	USA	546	Adults	Random	Religious denomination Religious attendance Religious application Religious commitment Religious identity Religious coping Religious social support		*√*	National Cancer Institute's Quick Food Scan	Conservative Protestant/others/no religion women more fat than Catholic women Males—none	Age, race, education, marital status, employment

16	Kim (2008) [87]	Texas, USA	424	Non-Hispanic Whites, 58–100 yrs	Random	Denomination Attendance Religious social support (% network in church & % network in religion)		*√*	Interview about fat reduction behavior	All R/S measures nonsignificant for men Women—Denomination nonsignificant; more % network in church, less fat reduction behavior; more % network in religion, more fat reduction behavior	Age, SES, urban-rural residence, living with someone, chronic illness, physically disabled, health & disability, general social support

17	McIntosh (1984) [88]	Virginia, USA	371	Elderly	Random	Religious participation Religious salience Religious disagreement Religious preference		*√*	24 hr dietary recall	+ve for salience and fat intake +ve for disagreement and fat intake More localistic, Methodists more fat, Brethren less fat	Sex, income

18	Schlundt (2008) [89]	Nashville, Tennessee, USA	3014	White & African Americans, >18 yrs	Stratified random	Religious denomination/affiliation Religious involvement Index (religious attendance, religiosity, perception of religion as a source of strength and comfort)	*√*	*√*	Eating Behavior Patterns Questionnaire Eating Styles Questionnaire (both Adapted from Behavioral Risk Factor Surveillance System)	Denomination—nonsignificant Religious involvement index positively associated with healthy eating behaviors and high-fat behaviors	Age, sex, race, education, income & employment

**Table:** (d) Degree of R/S studies

No.	First author (publication year)	Location	*N*	Population	Sampling method	R/S measures	Dietary measures		Dietary assessment methods	Finding	Control variables
							F/V	Fat			
19	Arredondo (2005) [90]	USA	211	Women, 18–65 yrs	Random	Church attendance	*√*	*√*	Block fat & fiber screener	Frequent churchgoers more fiber than nonchurchgoers Churchgoers (frequent & infrequent) more fiber than nonchurchgoers	Education, marital status, employment, age

20	Baruth (2011) [91]	South Carolina, USA	1136	African Americans, >18 yrs	Random	Perceived environmental church support (perceived written informational, perceived spoken informational, perceived instrumental, total perceived church support)	*√*	*√*	National Cancer Institute Fruit and Vegetable screener Servings of fruit and vegetable Fat- and Fiber- Related Behavior Questionnaire	More fruit and vegetable (i) more total perceived church support, perceived written informational support & perceived spoken informational support More low-fat behavior (i) more total perceived church support and perceived written informational support	Sex, years of education, health rating, age, BMI, influence of church

21	Benjamins (2012) [92]	USA	351	Jewish students, 5th–8th grades	Convenience	Religious beliefs & health	*√*		Youth Risk Behavior Survey (5 fruit and vegetable daily)	Nonsignificant	Gender, weight status, dieting, parental involvement, confidence

22	Debnam (2012a) [93]	USA	2370	African Americans, >21 yrs	Probability-based but not representative	Religion social support (emotional support received, emotional support provided, anticipated support, negative interaction)	*√*		National Cancer Institute's Five-A-Day Survey	More social support, more fruit and vegetable (i) additive effect: emotional religious support	Age, education, sex, self-rated health status

23	Debnam (2012b) [94]	USA	2370	African Americans, >21 yrs	Random	Spiritual Health Locus of Control Scale (active spiritual, passive spiritual)	*√*		National Cancer Institute's 5-A-Day Survey	Overall: +ve for active spiritual and daily fruit servings −ve for passive spiritual and daily vegetable servings Males: −ve for passive spiritual and daily vegetable servings; nonsignificant for fruit Females: −ve for passive spiritual and daily vegetable servings; none for fruit	Age, education, health status

24	Fife (2011) [95]	Northeast, USA	510	African American university students	Convenience	Duke University Religion Index	*√*		Youth Risk Behavior Survey (i) Ate no fruit during the past 7 days (ii) Ate no salad during the past 7 days (iii) Drank no 100% fruit juice during the past 7 days	Chi-square test: (i) nonsignificant—ate no fruit during the past 7 days; ate no salad during the past 7 days (ii) significant—drank no 100% fruit juice during the past 7 days Logistic regression: (i) univariate & multivariate—“intrinsic only” group more likely to drink no 100% fruit juice during the past 7 days	Year in school, gender

25	Franklin (2007) [96]	USA	1273	Adults, 18–96 yrs	Stratified random	Religious health fatalism questionnaire		*√*	Fat-increasing behavior Fat-decreasing behavior	High fatalism +ve associated with both high fat-increasing behavior & high fat-decreasing behavior	Age, race, gender, income, education

26	Friedlander (1985) [97]	Jerusalem, Israel	746	Jewish adults	Multistage random	Degree of religiosity (Orthodox, traditional, secular)	*√*	*√*	24-hr dietary recall	+ve for total fat, saturated fat, and P : S ratio in males Nonsignificant for fruit and vegetable intake	Age, region of origin, BMI, social class, seasonality

27	Hart (2004) [98]	USA	2375	Adults, >35 yrs	Random	Religious orientation (intrinsic versus extrinsic)	*√*	*√*	Fat- and Fiber- Related Behavior Questionnaire	Nonsignificant for fruit and vegetable More extrinsic, less fat	Age, sex, race, education, marital status, church size

28	Hart (2006) [99]	Seattle, USA	2375	Adults, >18 yrs	Random	Cohesiveness of religious organization members		*√*	Fat- and Fiber- Related Behavior Questionnaire	More cohesiveness, less fat, but nonsignificant after controlling for age and race	Age, race

29	Hart (2007) [100]	Washington, USA	1520	Adults, >18 yrs	Random	Social environmental (cohesion, leader support, order/organization, leader control)	*√*	*√*	Fat- and Fiber- Related Behavior Questionnaire	More cohesion, more order/organization, more fruit and vegetable More cohesion, order/organization, lower fat	Age, race, gender, education marital status, size of religious organization

30	Holt (2005) [101]	Missouri, USA	1227	African American women, 18–65 yrs	Convenience	Religiosity (beliefs & behavior) (i) 4 categories: low religious behavior only, belief only, high religious	*√*		National Cancer Institute's 5-a-day survey	Fruit/vegetable intake in descending order: Higher religious, behavior only, belief only, low religious	Education, income examined as potential covariates but not included because they are not associated with religious orientation

31	Lytle (2003) [102]	Minnesota, USA	3878	Adolescents	Random	Spiritual beliefs in health behaviors	*√*		Fruit and vegetable food frequency scale (from Behavioral Risk Factor Surveillance System)	Higher spiritual belief, more fruit and vegetable	Demographic & psychosocial variables

32	Obisesan (2006) [103]	USA	14,094	Nonpregnant adults, >20 yrs	Multistage random	Church attendance		*√*	FFQ 24-hr dietary recall	Nonsignificant	Age, sociodemogra-phic variables, health status

33	Park (2009) [104]	Hartford Hospital, USA	167	Cancer survivors, 18–55 yrs	Convenience	Religious service attendance Daily spiritual experiences Religious struggle Spiritual strain scale Mediator: Self-assurance	*√*		5 servings of fruit and vegetable a day	Daily spiritual experiences positively related to daily 5 servings of fruit and vegetable Self-assurance not related to 5 servings of fruit and vegetable, so no mediation test conducted	No

34	Pitel (2012) [105]	Slovakia	3674	Adolescents	Stratified random	Religiosity (religious attendance & self-rated importance of religious faith)	*√*		No regular fruit and vegetable consumption	Nonsignificant	Age, parental divorce, parental education, family affluence, degree of urbanization, ethnicity

35	Reeves (2012) [106]	Jackson, USA	2378	African Americans	Random	Organized religious activity Private prayer Daily spiritual experiences		*√*	FFQ—% calories from fat	Nonsignificant	None

36	Salmoirago-Blotcher (2011) [107]	USA	71,689	Postmenopausal women, 50–79 yrs	Random	Service attendance		*√*	FFQ—saturated fat intake	Nonsignificant	Age, race, marital status, education, health insurance, enrollment status, physical functioning, self-rated health

37	Shmueli (2007) [108]	Israel	3056	Jews, >18 yrs	Random	Religiosity—secular, observant, religious	*√*		FFQ	Nonsignificant	Age, gender, education, marital status, ethnic origin, socioeconomic status

38	Underwood (2006) [109]	Midwest, USA	471	African Americans, >20 yrs	Convenience	Religious intensity/religiousness Spiritual intensity/spirituality Religious practices	*√*	*√*	Public health Service health Style self-test	Very/moderately spiritual, very/moderately religious, more collaboration—less fruit and vegetable, more fat	None

39	Wiist (2012) [110]	Web-based	811	Buddhists, >18 yrs	Convenience	Buddhist Devoutness Index (Buddhist practices & beliefs)	*√*	*√*	FFQ	Nonsignificant	Age, gender, income, disability, social support

Abbreviations:

F/V: fruit/vegetable.

FFQ: food frequency questionnaire.

P : S:polyunsaturated fat : saturated fat.

**Table 2:** 

Categories of R/S measures	Paper no. in Table 1
Institutional	
Attendance	15, 16, 19, 32, 33, 36
Organized religious activity	35
Perceived environmental church support	20
Religious social support	15, 16, 22
Cohesiveness of religious organization members	28
Social environment	29
Religious identity	15

Ideological	
Religious beliefs and health	21
Religious application	15
Religious coping	15
Spiritual belief in health behaviors	31
Religious salience	17
Religious disagreement	17
Religious problem-solving	38
Religious struggle	33
Spiritual health locus of control	23
Religious health fatalism	25

Private devotion	
Private prayer	35
Religious orientation (intrinsic versus extrinsic)	27

Spiritual	
Daily spiritual experience	33, 35

Multidimensional	
DUREL	24
Religiosity (beliefs and practices)	30
Religious participation	17
Religiosity (attendance and self-rated importance)	34
Religious involvement index	18
Jewish religiosity	26, 37
Religious commitment	15
Buddhist devoutness index	39

Generic	
Religious intensity	38
Spiritual intensity	38

**Table 3:** Dietary assessments of fruit and vegetable and fat intake.

Dietary assessment methods	Fruit and vegetable		Fat	
	Total	%	Total	%
Dietary records	0	0	2	7.4
24-hour dietary recall	2	6.9	6	22.2
Food frequency	8	27.6	9	33.3
Brief dietary assessments	19	65.5	10	37.0
Dietary history	0	0	0	0

Total	29	100.0	27	100.0

**Table 4:** Categories of R/S measures and fruit and vegetable intake.

Categories	Relationships				Total	%
	+	−	Mixed	None		
Institutional	4	0	0	1	5	26.3
Ideological	1	1	1	1	4	21.1
Personal devotion	0	0	0	0	0	0.0
Existential	1	0	0	0	1	5.3
Multidimensional	2	0	1	4	7	36.8
Generic	0	0	1	1	2	10.5

Total	8	1	3	7	19	100

Mixed: when an R/S measure is positively associated with fruit intake and negatively associated with vegetable intake or vice versa, or when a R/S measure is positively/negatively associated with fruit intake and not associated with vegetable intake or vice versa.

**Table 5:** Categories of R/S measures and total fat intake.

Categories	Relationships				Total	%
	+	−	Mixed	None		
Institutional	2	0	1	8	11	40.7
Ideological	0	3	1	2	6	22.2
Personal devotion	1	0	0	1	2	7.4
Existential	0	0	0	1	1	3.7
Multidimensional	0	2	0	3	5	18.5
Generic	0	2	0	0	2	7.4

Total	3	7	2	15	27	100

Positive (+) relationship: a higher score of R/S measure is associated with *lower* fat intake; negative relationship (−): a higher score of R/S measure is associated with *higher* fat intake.

Mixed: when an R/S measure is both positively and negatively associated to fat intake.

**Table 6:** Categories of studies.

Dietary intake	Relationships				Total
	+	−	Mixed	0	
Fruit and vegetable	9	1	1	6	17
Fat	3*	3*	2	7	15

Notes: *positive (+): a higher score of R/S measure is associated with *lower* fat intake; negative (−): a higher score of R/S measure is associated with *higher* fat intake.
